# Structural basis and functions of abscisic acid receptors PYLs

**DOI:** 10.3389/fpls.2015.00088

**Published:** 2015-02-19

**Authors:** Xing L. Zhang, Lun Jiang, Qi Xin, Yang Liu, Jian X. Tan, Zhong Z. Chen

**Affiliations:** ^1^Department of Pediatrics, Affiliated Hospital of Guangdong Medical CollegeZhanjiang, China; ^2^State Key Laboratory of Agrobiotechnology, College of Biological Sciences, China Agricultural UniversityBeijing, China; ^3^National Center for Nanoscience and TechnologyBeijing, China

**Keywords:** abscisic acid, PYR/PYL/RCAR, PP2Cs, SnRK2s, ABA analogs, crystal structures

## Abstract

Abscisic acid (ABA) plays a key role in many developmental processes and responses to adaptive stresses in plants. Recently, a new family of nucleocytoplasmic PYR/PYL/RCAR (PYLs) has been identified as *bona fide* ABA receptors. PYLs together with protein phosphatases type-2C (PP2Cs), Snf1 (Sucrose-non-fermentation 1)-related kinases subfamily 2 (SnRK2s) and downstream substrates constitute the core ABA signaling network. Generally, PP2Cs inactivate SnRK2s kinases by physical interaction and direct dephosphorylation. Upon ABA binding, PYLs change their conformations and then contact and inhibit PP2Cs, thus activating SnRK2s. Here, we reviewed the recent progress in research regarding the structures of the core signaling pathways of ABA, including the (+)-ABA, (−)-ABA and ABA analogs pyrabactin as well as 6AS perception by PYLs, SnRK2s mimicking PYLs in binding PP2Cs. PYLs inhibited PP2Cs in both the presence and absence of ABA and activated SnRK2s. The present review elucidates multiple ABA signal perception and transduction by PYLs, which might shed light on how to design small chemical compounds for improving plant performance in the future.

## Introduction

### Abscisic acid

Abscisic acid (ABA) was discovered half a century ago (Addicott and Lyon, 1969; Milborrow, 1974; Cutler et al., 2010). Briefly, several groups isolated from different plant tissues plant growth regulators that could promote leaf abscission (Ohkuma et al., 1963) and seed dormancy (Cornforth et al., 1965b) and inhibit growth (Bennet-Clark and Kefford, 1953) and embryo germination (Cornforth et al., 1965b). Chemical analyses demonstrated that the activities of the isolated endogenous extracts were exerted by the same compound, which was ultimately named abscisic acid (Cornforth et al., 1965b; Milborrow, 1967).

ABA is an important sesquiterpenoid phytohormone that is derived from isopentenyl pyrophosphate (Nambara and Marion-Poll, 2005), and its chemical structure was finally confirmed by spectroscopic methods (Ohkuma et al., 1965) and chemical synthesis (Cornforth et al., 1965a). ABA is a pivotal regulator in plants and coordinates a complex regulatory network enabling plants to cope with abiotic stresses, such as drought, salinity, and temperature fluctuations (Verslues et al., 2006; Cutler et al., 2010; Kim et al., 2010; Miyakawa et al., 2013). Generation of ABA by cleavage of ABA conjugates (Lee et al., 2006) or *de novo* ABA biosynthesis (Nambara and Marion-Poll, 2005) significantly increases the ABA content under abiotic stress and thus regulates gene expression to assist plants in adapting to adverse environmental conditions (Hetherington, 2001; Schroeder et al., 2001). ABA also plays a key role in plant growth and development under non-stress conditions, including during embryo, seed and seedling development (Finkelstein et al., 2002) and seed dormancy (Finkelstein et al., 2008).

### Identification of ABA receptors

**Figure 1:**
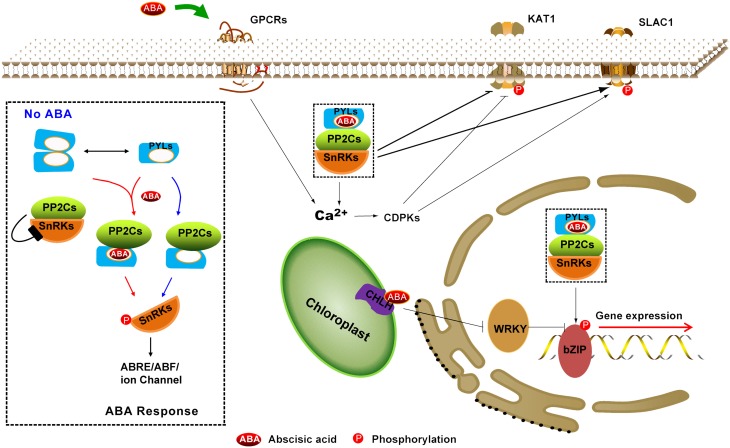
**Summary of the ABA perception and signaling pathway**. ABA receptors include nucleocytoplasmic PYR/PYL/RCARs (PYLs), most likely the plastid-localized Mg-chelatase H subunit (CHLH/GUN5/ABAR) and plasma membrane–localized GPCR type G-proteins (GPCRs). A core signaling pathway (black dotted rectangle enlarged in left) consists of PYLs, PP2Cs, SnRK2s, and downstream substrates, which control gene expression (long ABA signaling pathway) and stomatal closure (short ABA signaling pathway). Dimeric PYLs cannot bind to PP2Cs in the absence of ABA; thus, SnRK2s activity is inhibited by PP2Cs. Nevertheless, a subfamily of monomeric PYLs exhibits constitutive inhibitory activity on PP2Cs. In the presence of ABA, PYLs contact and inhibit PP2Cs, leading to the activation of SnRK2s through autophosphorylation. Then, the activated SnRK2 targets ABA-responsive element binding factors (such as bZIP) to regulate gene expression in the nucleus and the cation channel SLOW ANION CHANNEL-ASSOCIATED 1 (SLAC1) and POTASSIUM CHANNEL IN *ARABIDOPSIS THALIANA* 1 (KAT1) to cause stomatal closure in cytoplasm. The solid line with double-headed arrows indicates the equilibrium between dimeric PYLs and monomeric PYLs. The solid line with an arrow indicates direct positive interactions. The solid line with a bar indicates repression.

There are usually three common features of a receptor and its ligand: high affinity, high specificity, and a saturable and reversible interaction. Conventional genetic screening is beneficial to plant hormone research. Many key components that are involved in hormone signaling pathways were identified by screening of *Arabidopsis* mutants with increased or decreased sensitivity to a hormone (Santner and Estelle, 2009). However, such genetic screening failed to identify ABA receptors. This failure is mainly attributed to functional redundancy or pleiotropic effects, including embryo or gamete lethality for ABA receptors (Santiago et al., 2012). On the other hand, biochemical techniques were widely used to identify ABA-binding proteins. ABAP1 in barley aleurone was first reported to be an ABA binding protein (Razem et al., 2004). However, the homologous FCA in *Arabidopsis*, an RNA-binding protein that is responsible for flowering time (Razem et al., 2006), was unsuccessful in reproducing the ABA-binding ability using radioligand binding assays (Risk et al., 2008). The filter-based ligand-binding assay that was adopted in the FCA might be artificial due to non-specific binding (Risk et al., 2008). Then, the Mg-chelatase H subunit CHLH/GUN5/ABAR from *Arabidopsis* (Shen et al., 2006) as well as its homologue from *Vicia faba* (Zhang et al., 2002) were identified as ABA-binding proteins. The overexpression of either the full-length (Shen et al., 2006) or the C-terminal half of CHLH in *Arabidopsis* showed a hypersensitivity to ABA (Wu et al., 2009). However, the homologous CHLH protein in barley refused to bind ABA (Muller and Hansson, 2009). CHLH affected ABA signaling in stomatal guard cells, but no obvious ABA binding was detected using radioligand binding assays (Tsuzuki et al., 2011). Further experiments are required to determine the functions of CHLH in the ABA signaling pathway (Figure 1). In addition to the above two proteins, pharmacological evidence suggested that GTG1/GTG2 (Pandey et al., 2009) and GCR2 (Liu et al., 2007) were also ABA-binding proteins. However, *Arabidopsis gtg1/gtg2* double mutants only slightly impaired the sensitivity to ABA in seed germination and stomatal responses (Pandey et al., 2009). The subsequent measurements were unable to detect the binding of ABA to GCR2 (Risk et al., 2009).

Our knowledge of ABA receptors was not clear until the major breakthrough of PYR/PYL/RCAR (hereafter referred to as PYLs) in 2009. Chemical genetics was used to find *PYRABACTIN RESISTANCE 1* (PYR1) mutants that were insensitive to the synthetic selective ABA agonist pyrabactin (Park et al., 2009). Meanwhile, the function of the family as an ABA receptor was confirmed by a yeast two-hybrid assay using the ABI1/2 or HAB1 as bait (Ma et al., 2009). Nine independent members of the PYLs family were identified as the major *in vivo* interactors of ABI1 (Nishimura et al., 2010). PYL8 plays a non-redundant role in the regulation of root ABA sensitivity (Antoni et al., 2013) and promotes lateral root growth by enhancing the MYB77-dependent transcription of auxin-responsive genes (Zhao et al., 2014). In addition to PYLs, there may be other intracellular and extracellular ABA receptors that need to be identified (Cutler et al., 2010; Klingler et al., 2010).

### The core signaling network in the ABA response

Recent studies have revealed the core ABA signaling components including ABA receptors (PYLs), type 2C protein phosphatases (PP2Cs), protein kinases [Snf1 (Sucrose-non-fermentation 1)-related kinases subfamily 2, SnRK2s] and downstream targets (Fujii et al., 2009; Umezawa et al., 2009) (Figure 1). When plants are challenged by various abiotic and biotic stresses, the endogenous ABA content increases and then exquisitely initiates some cellular signaling network to switch on adaptive responses and to regulate numerous developmental processes. ABA binds to PYLs and then the binary complex physically interacts with PP2Cs. The PYLs-PP2Cs heterodimer precludes substrate SnRK2s binding to PP2Cs, and thus stimulates SnRK2s kinase activity, which was formerly inhibited by PP2Cs (Yoshida et al., 2006; Park et al., 2009; Santiago et al., 2009b; Umezawa et al., 2009) (Figure 1). Activated SnRK2s can target NADPH oxidases (Sirichandra et al., 2009) and ion channels, such as the SLOW ANION CHANNEL-ASSOCIATED 1 (SLAC1) (Geiger et al., 2009; Sirichandra et al., 2009; Vahisalu et al., 2010) and the K^+^ channel in *Arabidopsis Thaliana* 1 (KAT1) (Sato et al., 2009), to control stomatal closure. In addition, activated SnRK2s can also target ABA-INSENSITIVE 5 (ABI5) (Nakashima et al., 2009a) and ABA-responsive element (ABRE) binding protein (AREB)/ABRE-binding factor (ABF) as well as transcription factors (TFs) (Furihata et al., 2006; Fujii and Zhu, 2009; Fujii et al., 2009; Fujita et al., 2009, 2013; Nakashima et al., 2009a) to regulate ABRE-dependent gene expression (Fujita et al., 2011, 2013) in seeds or vegetative tissues. So far, 14 members of PYLs, six to nine group-A PP2Cs, three subclass III SnRK2s and four to nine group-A bZIP TFs are involved in the core ABA signaling pathway (Klingler et al., 2010; Hauser et al., 2011; Takezawa et al., 2011), which was successfully reconstituted *in vitro* (Fujii et al., 2009) (Figure 1).

Since 2009, a rapidly growing body of literature has supported a double negative regulatory system that orchestrates PYLs as *bona fide* ABA receptors, PP2Cs as key negative regulators and SnRK2s as positive regulators, which play a critical role in ABA signaling networks (Cutler et al., 2010; Hubbard et al., 2010; Melcher et al., 2010b; Raghavendra et al., 2010; Umezawa et al., 2010; Joshi-Saha et al., 2011; Santiago et al., 2012; Miyakawa et al., 2013). As ABA receptors, PYLs have witnessed abundant structural and functional data from independent research groups (Melcher et al., 2009; Miyazono et al., 2009; Nishimura et al., 2009; Santiago et al., 2009a; Yin et al., 2009; Hao et al., 2010, 2011; Peterson et al., 2010; Yuan et al., 2010; Sun et al., 2012; Zhang et al., 2012, 2013; Li et al., 2013). In this review, we will predominantly focus on the structural data regarding the perception and recognition of (+)-ABA, (−)-ABA or ABA analogs by PYLs, their inhibitory interaction with PP2Cs, the autoactivation of SnRK2s through autophosphorylation and PP2Cs inhibiting SnRK2s through physical binding as well as mutual packing of their catalytic sites and dephosphorylation.

## Structures of PYLs receptores

### Apo-form of PYLs

**Figure 2:**
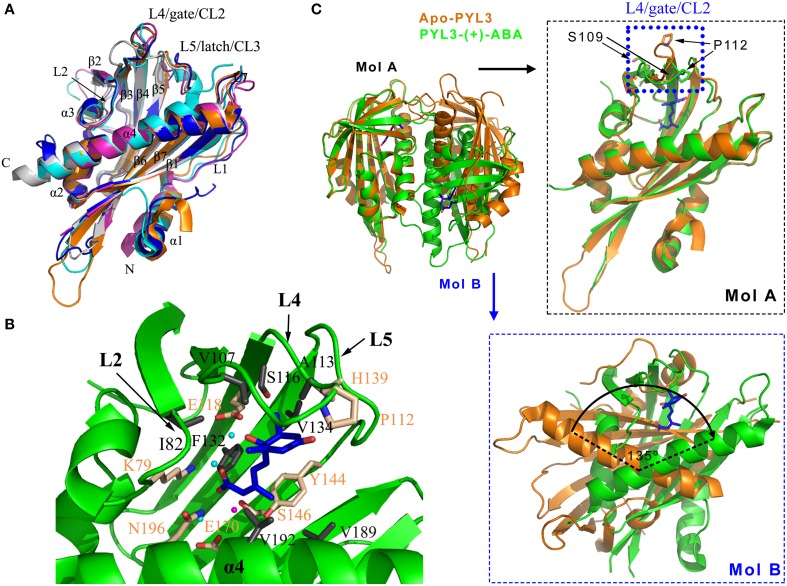
**(+)-ABA induced conformation changes of PYLs. (A)** PYR1 (PDB ID: 3K90, cyan), PYL1 (PDB ID: 3KAY, magenta), PYL2 (PDB ID: 3KDH, blue), PYL3 (PDB ID: 3KLX, orange), and PYL10 (PDB ID: 3R6P, gray) are shown in cartoon. **(B)** The ligand-binding pocket and the residues partaken in the hydrophobic and polar interactions of (+)-ABA-bound PYL3 (PDB ID: 4DSC, green). The ligand-binding pocket is encompassed by the L2, L4, L5 loops and α4 helix. The involved hydrophobic and polar residues were shown in gray and wheat sticks, respectively. Three water molecules involved in hydrogen bonds and one Mg^2+^ are shown in cyan and magenta spheres, respectively. 2D map of these interactions were seen in Figures S1, S2. **(C)** Superposition of *cis*-dimeric apo-PYL3 and trans-dimeric PYL3-(+)-ABA. The P112 residue moves toward the pocket to close the “gate” loop, whereas the S109 residue is flipped outward the cavity in response to (+)-ABA.

There are 14 PYLs family members, named PYR1 and PYL1-PYL13 in *Arabidopsis*, that belong to the START superfamily (Iyer et al., 2001). Although several structural homologs, such as the pollen allergen Bet V 1α and CSBP from *Vigna radiata*, were previously known, it remained unclear how receptors perceive the ABA molecule. To date, the crystal structures of PYR1 (Nishimura et al., 2009; Santiago et al., 2009a), PYL1 (Miyazono et al., 2009), PYL2 (Melcher et al., 2009; Yin et al., 2009), PYL3 (Zhang et al., 2012, 2013), PYL5 (Zhang et al., 2013), PYL9 (Zhang et al., 2013; Nakagawa et al., 2014), PYL10 (Hao et al., 2011; Sun et al., 2012), and PYL13 (Li et al., 2013) have been reported (Figure 2A). From the published apo-PYLs structures, we know that all PYLs share a highly similar helix-grip structure that is characterized by a seven-stranded β-sheet that is flanked by two α-helices. In addition, a particular feature of PYLs receptors is an α-helix in the N termini.

### (+)-ABA-bound PYLs

Based on ABA-bound structures, such as PYR1 (Nishimura et al., 2009; Santiago et al., 2009a), PYL1 (Miyazono et al., 2009), PYL2 (Melcher et al., 2009; Yin et al., 2009), PYL3 (Zhang et al., 2012), PYL9 (Zhang et al., 2013), and PYL10 (Hao et al., 2011; Sun et al., 2012), These structural architectures share a common ligand-binding pocket. The L2 loop between the α3 helix and β2 strand, the L4 loop (also referred to as CL2 or “gate” loop) between the β3 and β4 strands, the L5 loop (also referred to as CL3 or “latch” loop) (Melcher et al., 2009; Yin et al., 2009) between the β5 and β6 strands, and the C-terminal helix α4 encompass the entrance of the ligand-binding pocket (Figure 2B, Figure S2), which is very important for ABA binding. PYLs possess a large internal cavity in which the ABA molecule sits by a combination of ionic bonds, hydrophobic interactions and water-mediated hydrogen bonds (Figure 2B, Figures S1, S2). The carboxyl of ABA forms a salt bridge with the amine group of lysine (PYR1 K59, PYL1 K86, PYL2 K64, PYL3 K79, PYL9 K63, and PYL10 K56) as well as a water-mediated hydrogen bond network with several side chains of polar residues. The addition of a bulky group to the carboxylic group of ABA is likely to interfere with the binding to PYLs. In this scenario, the carboxylic group of ABA coupled to the amino group of a 10-atom spacer arm of a sepharose resin was utilized to identify ABA binding proteins and an ABA receptor such as CHLH (Zhang et al., 2002; Shen et al., 2006). Such approach probably cripples the interaction between the carboxylate group of ABA and ABA binding proteins or receptor. Therefore, it is suggested that ABA binding by CHLH must be further confirmed (Santiago et al., 2012). Moreover, the hydroxyl group and the ketone group of ABA also interact with the polar side chains of PYLs through water-mediated hydrogen bonds. In addition, the pentadienoic acid moieties and the cyclohexene contact hydrophobically with many inward-facing apolar side chains in the PYLs' cavity. These residues that are involved in binding ABA are strictly conserved in PYLs.

The superposition of the apo-PYLs and ABA-bound PYLs made it possible to depict the ABA-induced conformational changes. Here, the superposition of the ABA-bound and apo-PYL3 shows obvious conformational changes in the two conserved loops that flank the entrance to the ABA binding pocket and the C-terminal α-helix (Melcher et al., 2009; Nishimura et al., 2009; Santiago et al., 2009a; Yin et al., 2009; Zhang et al., 2012). When ABA binds to the receptor, the P112 residue of PYL3 on the “gate” loop (PYR1 P88, PYL1 P115, and PYL2 P92) moves toward the pocket to close the “gate” loop, whereas the S109 residue on the “gate” loop (PYR1 S85, PYL1 S112, and PYL2 S89) is flipped outward the cavity (Figure 2C right panel). In addition, the imidazole group of the H139 residue on the “latch” loop (PYR1 H115, PYL1 H142, and PYL2 H119) orientates inward the cavity to contact ABA. In addition, the α-helix α4 in the C termini moves slightly toward ABA to facilitate the closure of the “gate” and “latch” loops. These altered conformations create a new surface that is favorable for binding to PP2Cs, which in turn lock the “gate” and “latch” loops into the closed conformation (discussed below). Thus, ABA perception by PYLs allosterically regulates the conformation of the “gate” and “latch” loops to switch the ABA signal transduction.

The structural changes by ABA perception that were mentioned above only considered one protomer of PYLs. Interestingly, based on the oligomeric state of apo-PYLs, the results from gel filtration chromatography, small angle X-ray scattering, static light scattering or analytical ultracentrifugation convincingly showed that PYL4-10, except for uncharacterized PYL7, are monomers, whereas PYR1 (Nishimura et al., 2009; Santiago et al., 2009a) and PYL1-3 are homodimers in solution (Dupeux et al., 2011b; Hao et al., 2011; Zhang et al., 2012). For homodimeric PYR1 and PYL1-2, the relative orientation of one protomer with respect to the other is slightly changed in response to ABA. As a result, a significant rearrangement of the interface is generated, leading to a diminished number of van der Waals contacts and hydrogen bonds, and consequently a weakening of the dimer interface (Nishimura et al., 2009; Yin et al., 2009). Similarly, *cis*-homodimer of PYL3 has also been observed in the apo-PYL3 structure (Figure 2C). However, ABA-bound PYL3 transforms into a *trans*-homodimer by one protomer rotation of almost 135° compared to the *cis*-homodimer (Figure 2C lower panel). The “gate” and “latch” loops in the *trans*-homodimer are more exposed in the solvent (Zhang et al., 2012). These data suggest that the binding of ABA could influence the dimer interface and the relative orientation of the two protomers from dimeric PYLs, which may be used to strictly regulate the ABA-dependent switching of signal transduction by PYLs (Umezawa et al., 2010).

## The stereospecificity of PYLs to (+/−)-ABA stereoisomers

**Figure 3:**
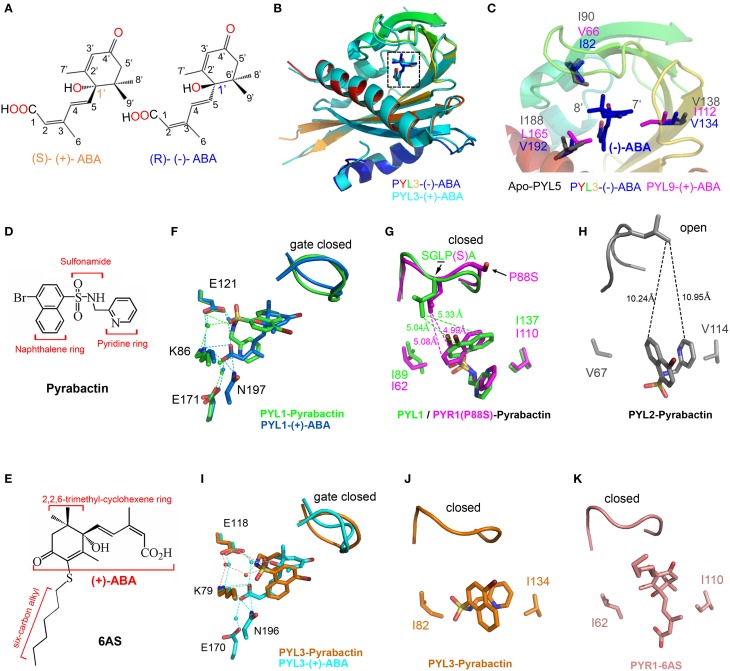
**Structural basis for the selective activation of ABA receptors by different ligands. (A)** Chemical structures of (+)-ABA and (−)-ABA. **(B)** Superposition of PYL3-(−)-ABA (PDB ID: 4JDA, rainbow) and PYL3-(+)-ABA (PDB ID: 4DSC, cyan). (−)-ABA and (+)-ABA were shown in blue and cyan sticks, respectively. **(C)** Superposition of PYL3-(−)-ABA (rainbow cartoon), PYL9-(+)-ABA (magenta sticks), and apo-PYL5 (gray sticks) indicates that the major variant residues underlie the stereospecificity of PYLs to (−)-ABA. Two bulk side chains of I112 and L165 in PYL9 seriously clash with the 7′ and 8′ methyl groups of (−)-ABA, respectively. The chemical structures of pyrabactin **(D)** and 6AS **(E)**. **(F–K)** Superposition of PYLs-ligands. Superposition of PYL1-pyrabactin (PDB ID: 3NEF, green) with PYL1-(+)-ABA (PDB ID: 3JRS, marine) shows the basis of pyrabactin as an agonist for PYL1, inducing the closure of the “gate” loop just as (+)-ABA. There are two residues determining the agonism and antagonism of pyrabactin for PYLs [PYR1(P88S)-pyrabactin (PDB ID: 3NJO, magenta) and PYL2-pyrabactin (PDB ID: 3NR4, gray)]. In particular, the superposition of PYL3-pyrabactin (PDB ID: 3OJI, orange) with PYL3-(+)-ABA (PDB ID: 4DSC, cyan) indicates that the “gate” loop of PYL3 closes in response to pyrabactin, which is not compatible for the insertion of the conserved tryptophan of PP2Cs. Therefore, pyrabactin is an antagonist for PYL3 (Zhang et al., 2012). The PYR1-6AS (PDB ID: 3WG8, wheat) structure shows that 6AS, mimicking (+)-ABA, induces the same conformation of the “gate” loop, but its long 3′ alkyl chains impedes PP2Cs docking; thus, 6AS is also an antagonist for PYLs (Takeuchi et al., 2014). All dotted lines represented the distance between two resides in angstrom units.

ABA contains one optical center at C1′, and the form of S-(+)-ABA exists in nature [hereafter referred to as (+)-ABA]. Another feature is the side chain of the ABA molecule, which contains two double bonds that are conjugated to the carboxylic acid. The configuration of the double bond that is adjacent to the ring is *trans*, while that proximal to the acid group is *cis* (Figure 3A). Upon exposure to UV light, biologically active 2-*cis*,4-*trans* ABA would be isomerized to the inactive form 2-*trans*,4-*trans* ABA (Cutler et al., 2010). In order to study the importance of the S-(+)-ABA stereoisomer and its biological activity, the unnatural but bioactive stereoisomer R-(−)-ABA was synthesized [hereafter referred to as (−)-ABA] (Lin et al., 2005; Zaharia et al., 2005) (Figure 3A). Both of the stereoisomers showed obviously different activities in many aspects, such as stomatal closure, but nonetheless a comparable activity in seed germination (Nambara et al., 2002; Xie et al., 2005). Microarray and genetic studies showed that the *in vivo* function of R-(−)-ABA also requires the signaling pathway of (+)-ABA (Xie et al., 2005; Huang et al., 2007). Two mechanisms were hypothesized to explain the bioactivity of (−)-ABA: one mechanism was that the same site was occupied between these two ABA stereoisomers by flipping the cyclohexene plane (Milborrow, 1974), and the other mechanism was the dual selectivity of ABA receptors (Nambara et al., 2002).

Generally, PYLs show a greater affinity for (+)-ABA than for (−)-ABA (Melcher et al., 2009; Miyazono et al., 2009). Although PYL9 shows a stringent preference for the natural (+)-CityABA, PYL5 can bind (−)-ABA with a K_*d*_ 8-fold higher than for (+)-ABA (Santiago et al., 2009b). (−)-ABA promoted the binding affinity of PYL2-4 but not PYR1 to HAB1 (Park et al., 2009). (−)-ABA could be structurally modeled into the PYL2 pocket without any steric clash (Yin et al., 2009). The initial 1.8 Å resolution structure was determined with mixed enantiomorphic (**+/−**)-ABA, suggesting that the chiral difference was accommodated within the ABA receptor binding pocket by the flipping of the ABA ring by 180° (Nishimura et al., 2009).

From the recent report, PYL5 showed the strongest binding affinity to (−)-ABA among all of the tested PYLs. PYL9 was a stringently exclusive (+)-ABA receptor (Zhang et al., 2013). PYL3 was a dual receptor to both ABA enantiomers. The structures of apo-PYL5, PYL3-(−)-ABA and PYL9-(+)-ABA were determined to elucidate the mechanism of stereospecificity of PYLs to ABA enantiomers (Zhang et al., 2013). Interestingly, the entire profile of (−)-ABA-bound PYL3 resembles that of PYL3-(+)-ABA, with a closed “gate” loop and a *trans*-dimeric rearrangement. The superimposition of these structures showed that the binding orientation of (−)-ABA in the PYLs pocket is obviously different from that of (+)-ABA (Figure 3B), which may deny the “flip” hypothesis. Structural and biochemical investigations showed that the major variable residues surrounding the mono-methyl and di-methyl groups of the ABA cyclohexene ring might underlay the preference of PYL binding to ABA enantiomers, such as steric hindrance by the two bulk side chains of I112 and L165 in PYL9 (Figure 3C). Moreover, the hydrophobic interaction through indirect interaction with the 8′, 9′ methyl groups of (−)-ABA (Figure 3C) also contributes to the stereospecificity of PYLs to ABA enantiomers because the V66I mutation increases the inhibitory ability of PYL9 to PP2Cs in the presence of (−)-ABA. The relative stereo specificity of different PYLs and their ability to bind alternative ligands could be used to explore the link between biochemical activities and physiological responses and may also provide useful tools to manipulate ABA signaling in both experimental and agricultural contexts (Hubbard et al., 2010).

## Pyrabactin as an agonist/antagonist of PYLs

Due to functional redundancy or pleiotropic effects (McCourt, 1999), the selective analogs of ABA are required to illuminate the function of a certain ABA receptor or find other unknown ABA receptors. Many analogs of ABA were designed mainly by substituting the 7′, 8′, or 9′-carbon atoms (Zaharia et al., 2005). Unlike ABA structurally, pyrabactin (Figure 3D), which was employed to find the first ABA receptor PYR1 by chemical genetics (Park et al., 2009), is a selective ABA agonist in seed germination and stomatal closure (Zhao et al., 2007; Puli and Raghavendra, 2012). Several years later, the structures of PYR1 and PYL1-3 complexed with pyrabactin were reported to clarify the selective activation of PYLs (Hao et al., 2010; Melcher et al., 2010b; Peterson et al., 2010; Yuan et al., 2010; Zhang et al., 2012). Pyrabactin as an agonist binds to PYL1 and PYR1 in a productive mode, whereas as an antagonist binds to PYL2 or PYL3 in a non-productive mode, indicating that pyrabactin can adopt different conformations in the conserved pocket of PYLs.

Structural analyses have revealed that pyrabactin lies in the cavity of PYL1 or PYR1 to induce the closure of the “gate” loop as ABA does (Hao et al., 2010; Peterson et al., 2010). Although pyrabactin structurally does not resemble ABA, several key interactions are conserved between PYL1-(+)-ABA and PYL1-pyrabactin (Hao et al., 2010; Melcher et al., 2010b) (Figure 3F). The pyridyl nitrogen of pyrabactin is located in the position of the carboxylate oxygen of ABA, forming water-mediated hydrogen bonds with the K86 and E171 in PYL1 (Figure 3F). In addition, the amine group of pyrabactin occupies the position of the hydroxyl group of ABA, which forms hydrogen bonds with N197 and E121 in PYL1. The sulfonamide of pyrabactin also forms hydrogen bonds with E121. In addition, the naphthalene ring of pyrabactin imitates the 2,6,6-trimethylcyclohexene ring of ABA, forming hydrophobic contacts with the “gate” loop to promote closure (Figure 3G). Moreover, the PYL1-pyrabactin-ABI1 complex structure reveals that pyrabactin inhibits PP2C activity in a productive mode (Melcher et al., 2010b).

In contrast, the binding of pyrabactin to the pocket of PYL2 does not provoke the closure of the “gate” loop (Melcher et al., 2010b; Peterson et al., 2010; Yuan et al., 2010) (Figure 3H). The orientation of pyrabactin occupying the pocket of PYL2 is rotated by approximately 90° compared to that in the pocket of PYR1 or PYL1. The naphthalene ring cannot induce the closure of the PYL2 “gate” loop because it is too far away to interact with each other (Figure 3H). The orientation of pyrabactin is positioned by the two small and hydrophobic residues V114 and V67 in PYL2, which correspond to I110 and I62 in PYR1 and I137 and I89 in PYL1, respectively (Figure 3G). Compared to the wild type, the V67I, V114I, and V67I/V114I mutants of PYL2 showed an increasing inhibition onto PP2Cs in response to pyrabactin (Peterson et al., 2010; Yuan et al., 2010). In addition, the I62V, I110V, and I62V/I110V mutants of PYR1 achieved only partial PP2C inhibition in response to pyrabactin (Peterson et al., 2010). These two smaller valine residues in PYL2 make the naphthalene ring deep in the pocket and far away from the “gate” loop, which results in pyrabactin as an antagonist binding to PYL2.

Intriguingly, the situation in PYL3 is totally different. The orientations of the naphthalene and pyridine ring of pyrabactin are significantly rotated compared to those in other PYLs-pyrabactin complexes. Moreover, the sulfonamide group moves to F81 and does not form a hydrogen bond with K79, while the conserved lysine residue in other PYLs-pyrabactin complexes forms a hydrogen bond with the pyridyl nitrogen to locate pyrabactin inside of the binding pocket. Pyrabactin can induce the closure of the “gate” in PYL3, but the “gate” loop moves further toward the binding pocket and tightly closes the “latch” (Figures 3I,J). The “gate” loop in PYL3 binds to pyrabactin tighter, which gives rise to the more compact space between the “gate” loop and the binding pocket compared to that in PYL1, PYL2, or PYR1. Therefore, there is no enough space between the “gate” loop and the “latch” loop for the insertion of conserved tryptophan residue from PP2Cs. Taken together, pyrabactin works as an antagonist for PYL3 (Figures 3I,J) (Zhang et al., 2012).

These different orientations of pyrabactin in PYLs either provide or impair interactions with the “gate” loop, inhibiting PP2Cs in a productive or non-productive mode, respectively. In addition, AM1, a small-molecule ABA mimic, acts as a potent activator of several members of PYLs (Cao et al., 2013), whereas 6AS, an ABA analog containing a six-carbon alkylsulfanyl that is linked to ABA's 3′ ring, was designed and confirmed as a potent ABA antagonist (Figures 3E,K) (Takeuchi et al., 2014). These reported structural data shed light on the concept of ABA receptor agonism and antagonism and are useful in the design of selective PYLs analogs.

## ABA-dependent inhibition of PP2Cs by PYLs receptors

### Architecture of the ternary complexes PYLs-(+)-ABA-PP2C

**Figure 4:**
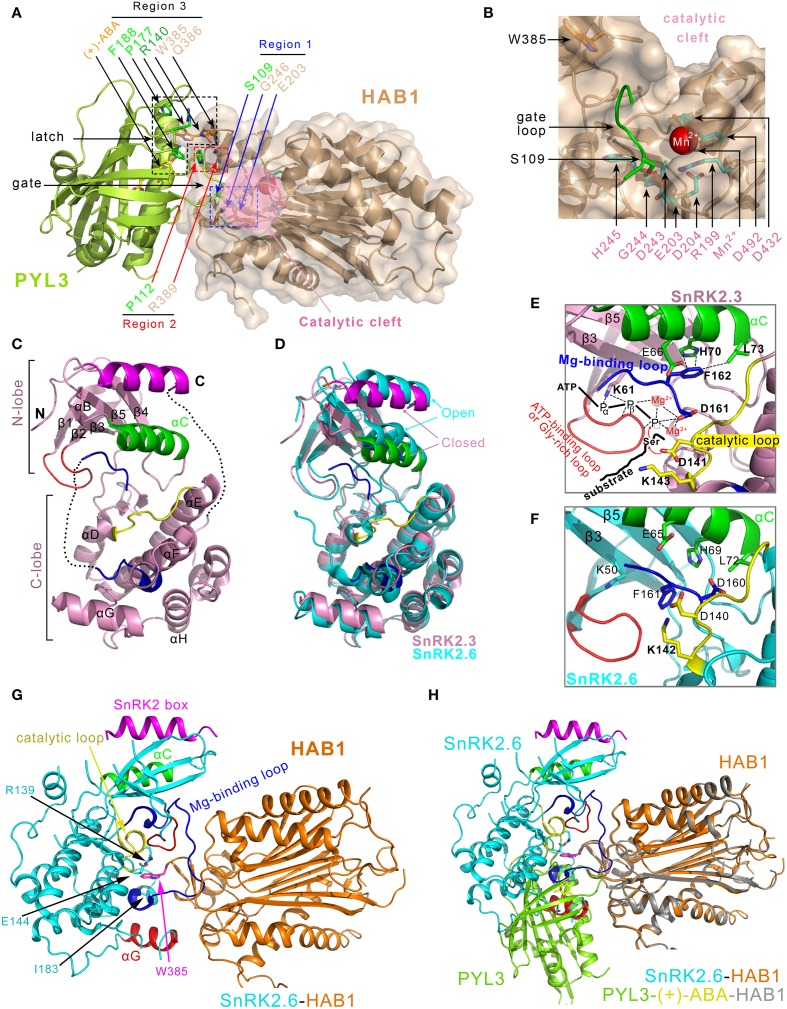
**Structural characterizations of the PYLs-(+)-ABA-HAB1 and SnRK2s-PP2Cs complex. (A)** Overview of three contact regions between PYL3 and HAB1 (PDB ID: 4DS8). The active site of HAB1 is shown in the pink circle. The PP2C contacts the S109 in the “gate” loop of PYL3 through its E203 and G246 in the active site cleft (Region 1), and contacts the P112 in the “gate” loop of PYL3 through its R389 residue (Region 2). The W385 and Q386 residues of PP2C (Region 3) contact several elements surrounding the entrance of the PYL3 cavity, including R140, P177, and F188 of PYL3 and the ketone group of ABA. **(B)** Details of the phosphatase active center, which consists of residues R199, E203, D204, D243, G244, H245, D432, and D492. Mn^2+^ is marked in a magenta sphere. **(C)** Structural characterization of SnRK2.3 (PDB ID: 3UC3) (Ng et al., 2011). Helix αC is displayed in green, the ATP-binding loop (Ng et al., 2011)/Gly-rich loop (Yunta et al., 2011) is displayed in red, the SnRK2 box (Ng et al., 2011)/DI domain (Yunta et al., 2011) is shown in magenta, the Mg^2+^ binding loop/activation loop are shown in blue, and the catalytic loop is colored yellow. **(D)** Superposition of the SnRK2.3 (see panel C) and SnRK2.6 (PDB ID: 3UC4, cyan) structures, which manifests that SnRK2.3 and SnRK2.6 adopt closed and open conformations, respectively. **(E)** SnRK2.3 adopts a partially active conformation. ATP, Mg^2+^ and substrate are introduced into apo-SnRK2.3 to illustrate active kinase conformational features. The residues that are involved in intra-molecular interaction network in the active sites are shown (Ng et al., 2011). **(F)** SnRK2.6 adopts an inactive conformation. E65, D160, and the catalytic D140 are far from the active site (Ng et al., 2011). **(G)** Overview of the SnRK2.6-HAB1 (PDB ID: 3UJG) complex structure. Three regions in SnRK2.6 are responsible for interaction with HAB1: the catalytic loop (yellow), the helix αG (red) and the Mg^2+^-binding loop (blue). In addition, R139, E144, and I183 (magenta) of SnRK2.6 establish interactions with the conserved W385 in HAB1 (cyan). **(H)** Overlay of the interaction surfaces from the SnRK2.6-HAB1 and PYL3-(+)-ABA-HAB1 complexes.

Four crystal structures of the ternary complexes PYL1-ABA-ABI1, PYL2-ABA-HAB1, PYR1-ABA-HAB1, and PYL3-ABA-HAB1 have been described (Melcher et al., 2009; Miyazono et al., 2009; Yin et al., 2009; Dupeux et al., 2011a; Zhang et al., 2012). PP2Cs dock into the ABA-bound PYLs, in which the major interface comprises the closed “gate” loop, the “latch” loop and the C-terminal helix (Figure 4A). These structures elucidate the mechanism by which PYLs change their conformation upon ABA binding to inhibit the phosphatase activity of PP2Cs in an ABA-dependent manner. In these structures, the PP2Cs catalytic cores (residues: ABI1 125-429, HAB1 172-511) adopt a fold with two central five-stranded β-sheets that are sandwiched by two pairs of α-helices, and the catalytic site is located at the edge of the two central β-sheets (Figure 4A). The N-terminal portion of the PP2Cs perhaps regulates PP2Cs activity via the recruitment of other interacting partners, but its structure has not been determined.

There are three characteristic interaction regions between the ABA receptors (PYL1-3) and the PP2Cs catalytic core (Figure 4A). First, the conserved serine residue in the “gate” loop (PYL1 S112, PYL2 S89, and PYL3 S109) establishes hydrogen bonds with the backbone amide of glycine (ABI1 G180 and HAB1 G246) and the metal-stabilizing carboxylic group of glutamic acid (ABI1 E142 and HAB1 E203) of PP2Cs (Figure 4A region 1 and Figure 4B). The involved glycine and glutamic acids are located at the catalytic site of PP2Cs; therefore, the catalytic site cleft of PP2Cs is blocked by the closed “gate” loop of PYLs, which is responsible for the inhibitory effect on the phosphatase activity. In addition, a conserved proline residue in the “gate” loop (PYL1 P116, PYL2 P93, and PYL3 P112) stacks with the guanidinium group of a conserved arginine residue in PP2Cs (ABI1 R304 and HAB1 R389) (Figure 4A region 2). This interaction promotes the “gate” loop enclosure and the insertion to the active site of PP2Cs. In addition, the indole ring of a conserved tryptophan residue (ABI1 W300 and HAB1 W385) inserts between the “gate” and “latch” loops of the ABA-bound PYLs (Figure 4A region 3). A representative water-mediated hydrogen network is established among the indole imine group of tryptophan, the ketone group of ABA, the backbone carbonyl of proline in the “gate” loop and the guanidinium group of arginine in the “latch” loop. Therefore, the phosphatase activity of PP2Cs is inhibited by interacting with ABA-bound PYLs. Furthermore, these PP2C-induced conformational changes of PYLs and the interaction between the conserved tryptophan of PP2C and ABA further stabilize the binding of ABA to PYLs and thus decrease the rate of ABA dissociation from the PYLs (Ma et al., 2009; Nishimura et al., 2009; Santiago et al., 2009b; Cutler et al., 2010). Consistently, the overexpression of the HAB1^W385A^ mutant in *Arabidopsis* leads to reduced ABA sensitivity (Dupeux et al., 2011a).

PYR1, PYL1, PYL2, and PYL3 are homodimers, while the PYLs-PP2Cs complexes are heterodimers in solution. The homodimeric interface of PYLs has a dramatic overlap with the heterodimeric interface of PYLs-PP2Cs. Therefore, the homodimeric PYLs must be dissociated into monomeric PYLs to bind to PP2Cs. ABA-binding could induce a conformational rearrangement of dimeric PYLs, which decreases the interface area and promotes the dissociation of dimeric PYLs (Yin et al., 2009; Zhang et al., 2012). It was postulated that PYLs exist *in vivo* as inactive homodimers and are incapable of binding or inhibiting PP2Cs (Yin et al., 2009). Therefore, the dimeric nature of PYLs may play some roles in the regulation of PYLs in plants, such as reducing the basal interaction between PYLs and PP2Cs.

## Dominant insensitivity of *abi1-1*, *abi2-1*, and *hab1-1* to ABA

The missense mutations *abi1-1* (G180D) and *abi2-1* (G168D) were isolated more than 20 years ago by genetic screenings in *Arabidopsis* to study the ABA signaling (Koornneef et al., 1984; Leung et al., 1994, 1997; Meyer et al., 1994; Rodriguez et al., 1998). ABI1 and ABI2 were negative regulators of ABA signaling, but both *abi1-1* and *abi2-1* mutants showed insensitive responses to ABA (Gosti et al., 1999; Merlot et al., 2001; Saez et al., 2004, 2006). The ABI1-1 protein cannot bind PYLs in the presence of ABA (Ma et al., 2009; Park et al., 2009) but retains the normal capacity to interact with SnRK2s (Umezawa et al., 2009; Vlad et al., 2009). The ternary complex structures of PYLs-(+)-ABA-PP2Cs showed that the conserved serine in the “gate” loop forms a hydrogen bond with the conserved glycine in the active site of PP2Cs (Figures 4A,B). The bulkier aspartic acid, which is substituted for the glycine, would disrupt the hydrogen bond, clash with the “gate” loop, and in turn impair the binding of PP2Cs (Yin et al., 2009).

## ABA-independent inhibition of PP2Cs by PYLs receptores

### ABA-responsive inhibition of PP2Cs

ABA first enters the hydrophobic pocket of PYLs, and then the ABA-bound PYLs bind to the downstream substrate PP2Cs, called the induced pathway (Ma et al., 2009; Park et al., 2009; Cutler et al., 2010). This mechanism is convincingly confirmed by the crystal structures of four PYLs-ABA-PP2Cs ternary complexes (Melcher et al., 2009; Miyazono et al., 2009; Yin et al., 2009; Dupeux et al., 2011a; Zhang et al., 2012). However, both yeast two-hybrid and in planta bimolecular fluorescence complementation experiments demonstrated that PYL9 and PYL5, in contrast to PYR1 and PYL1-4, show constitutive interaction with PP2Cs (Ma et al., 2009; Park et al., 2009; Santiago et al., 2009b; Szostkiewicz et al., 2010). ABI1 can be co-purified with PYL5–12 from *Arabidopsis* plants (Nishimura et al., 2010). Biochemical assays *in vitro* also showed that PYR1 and PYL1-3 inhibit PP2Cs (ABI1, HAB1, HAB2, and PP2CA) in an ABA-dependent manner, whereas PYL5-6 and PYL8-10 can inhibit PP2Cs even in the absence of ABA (Hao et al., 2011). Therefore, PYLs contact and inhibit PP2Cs, independent of ABA, called the constitutive pathway.

**Figure 5:**
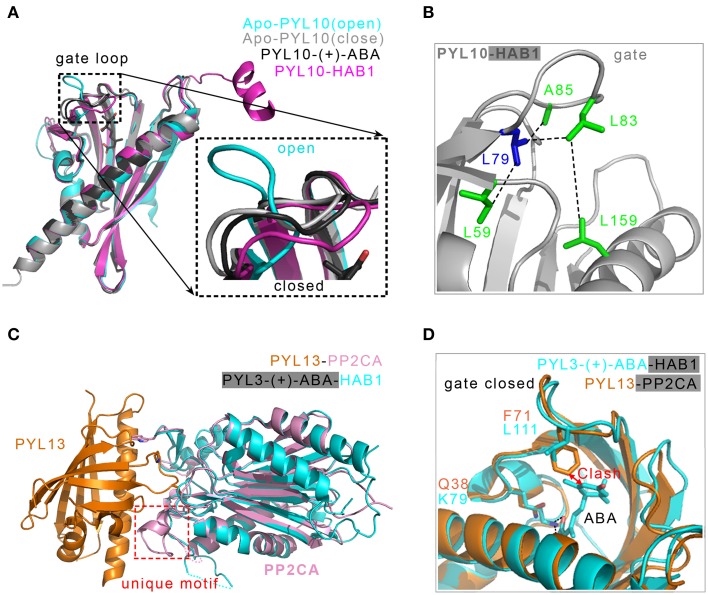
**Structural basis of the ABA-independent inhibition of PP2Cs by both PYL13-PP2CA and PYL10-HAB1 complexes. (A)** Structural superposition of two apo-PYL10 (PDB ID: 3RT2 and 3UQH), PYL10-(+)-ABA (PDB ID: 3R6P) and PYL10-HAB1 (PDB ID: 3RT0). A putative two-conformation dynamical equilibrium may exist (Sun et al., 2012); therefore, PYL10 displays a high tendency to inhibit PP2Cs in the absence of (+)-ABA (Hao et al., 2011). **(B)** Structural analysis of the constitutive inhibition of PP2Cs by PYL10. The bulkier hydrophobic residue L79 in PYL10, demarcating the β3 strand and the “gate” loop, provides a platform to dock the hydrophobic L83 in the “gate” loop and close it (Hao et al., 2011). **(C)** Two PP2Cs from PYL13-PP2CA (PDB ID: 4N0G) and PYL3-(+)-CityABA-HAB1 (PDB ID: 4DS8) are superimposed, the PYL3-(+)-ABA in PYL3-(+)-ABA-HAB1 is not displayed. The PP2CA-unique motif supplies a new interface with PYL13 (Li et al., 2013). **(D)** Two major events are responsible for the ABA-irresponsive and constitutive inhibition of PP2CA by PYL13. The F71 in the “gate” loop collides with the cyclohexene plane, and the Q38 in PYL13 substituting for the conserved lysine loses the essential salt bonds with the carboxylic group of (+)-ABA (Li et al., 2013). Two PP2Cs including HAB1 and PP2CA are not shown.

Recently, crystal structures of apo-PYL10 and the binary complex PYL10-HAB1 (Figure 5A) revealed the molecular mechanism by which a subclass of PYLs, represented by PYL10, inhibits PP2Cs even in the absence of ABA (Hao et al., 2011). The alignment of all PYLs primary sequences, particularly the residues that contact the “gate” loop, showed that the bulkier hydrophobic residue L79 in PYL10 provided a platform to dock the hydrophobic L83 in the “gate” loop, thus facilitating a closed conformation of the “gate” loop (Figure 5B). Nevertheless, the corresponding residues in all of the other PYLs except for PYL13 are occupied by a smaller valine. Structural-guided biochemical assays indicated that PYLs in a monomeric state are the first prerequisites for the ABA-independent inhibition of PP2Cs. Secondly, the residues guarding the entrance to the cavity of these PYLs should be bulky and hydrophobic (Hao et al., 2011). The apo-PYL10 with the “gate” loop in closed conformation was also determined, which builds a dynamic equilibrium model between the open and closed conformations of PYL10 (Sun et al., 2012). Comparing the structures of apo-PYL10 and other known apo-PYLs, the “gate” loop of the apo-PYL10 displayed the strongest tendency to form a closure conformation (Figure 5). Thus, ligand-free PYL10 is able to adopt a compatible conformation for PP2C recognition in the absence of ABA.

However, based on the two abovementioned principles for the constitutive inhibition of PP2Cs, the PYL2^V87L/I88K^ double- mutation, incorporating PYL10's unique “gate” sequence into the low basal-activity receptor PYL2, only partially augments the constitutive inhibition ability onto PP2Cs (Hao et al., 2011). To create constitutively active receptors, a recent study performed site-saturation mutagenesis at 39 residues in PYR1 that were involved in contacting ABA or PP2Cs, and triple or quadruple mutations (PYR1^H60P/V83F/F159V^, PYR1^H60P/V83F/M158I/F159V^) with full activation were finally obtained and were indistinguishable from ABA-saturated PYR1 (Mosquna et al., 2011). These mutations would be useful tools for studying the functions of individual PYLs *in vivo* (Mosquna et al., 2011; Miyakawa et al., 2013).

Although some monomeric PYLs can bind to PP2Cs in the absence of ABA, the inhibitory efficiency is obviously lower compared to that in the presence of ABA. The constitutive interaction between PYLs and PP2Cs, as well as endogenous ABA content alteration, is perhaps orchestrated to regulate ABA signaling for plant survival and growth (Santiago et al., 2009b; Hao et al., 2011).

### ABA-irresponsive inhibition of PP2Cs

There are 14 members of the PYLs family (Ma et al., 2009; Park et al., 2009). *In vivo* reconstitution assays in *Arabidopsis* protoplasts indicated that all of the PYLs except for PYL13 function as ABA receptors in the ABA signaling pathway (Fujii et al., 2009). It was reported that PP2Cs could physically interact with PYL13 (Joshi-Saha et al., 2011). Recently, one group reported that PYL13 can modulate the ABA pathway by interacting with and inhibiting both other PYLs and PP2Cs (Zhao et al., 2013). Nevertheless, PYL13 has an ABA receptor function by interacting with PP2C in early plant development (Fuchs et al., 2014). The PYL13-PP2CA complex structure elucidated the molecular basis for the specific interaction between PP2CA and PYL13 (Li et al., 2013). PYL13-PP2CA shares the common binding features of other PYLs and PP2Cs in the presence of (+)-ABA. In addition, the unique motif of PP2CA, including a short helix and a loop, is not found in ABI1 and ABI2 and is different from that in HAB1 and HAB2. This unique motif supplies a new interface with PYL13 mainly through direct and water-mediated hydrogen bonds (Figure 5C). The known structures demonstrated that a conserved lysine residue in the PYLs pocket is required to anchor the carboxylate group of ABA (Melcher et al., 2009; Miyazono et al., 2009; Nishimura et al., 2009; Santiago et al., 2009a; Yin et al., 2009; Zhang et al., 2012). However, the alignment of all of the PYLs sequences showed that the conserved lysine is substituted with the Q38 at the corresponding position of PYL13, which cripples the essential link with ABA. In addition, structural superimposition showed that there would be a steric clash between the aromatic ring of the F71 residue in PYL13 and the hydrophobic moiety of ABA. This clash can be completely abrogated by the residue leucine at the corresponding position of other PYLs (Li et al., 2013) (Figure 5D). Neither the Q38K nor F71L single mutation could endow PYL13 with response to ABA. The double mutation Q38K/F71L converted PYL13 into an ABA-dependent inhibitor to all of the tested PP2Cs, including PP2CA, ABI1, HAB1, and HAB2. Therefore, the lack of the conserved lysine in the pocket and the replacement of leucine with phenylalanine in the “gate” loop account for the ABA irresponsiveness of PYL13 (Li et al., 2013) (Figure 5D).

Although PYL13 and PYL10 show a high basal activity of inhibiting PP2Cs, both are different in response to ABA. PYL13 does not bind to ABA because of steric hindrance and the absence of the conserved lysine residue. PYL10 has the capacity to interact with ABA, and the potent constitutive inhibition of PYL10 onto PP2Cs can be further enhanced by ABA application. In addition, PYL13 antagonized PYL10 in the ABA-independent inhibition of PP2Cs because they were the only two PYLs with a leucine at the demarcation point of the β3 strand and the “gate” loop, facilitating the closure of the “gate” loop (Hao et al., 2011; Li et al., 2013).

The constitutive inhibition of PP2Cs can be achieved by the ABA-irresponsive receptor (such as PYL13) or ABA-responsive receptor (such as PYL10). These two types of receptors together with ABA-dependent receptors (such as PYR1 and PYL1-3) may provide a quantitative, fine-tuned and sensitive regulation in the ABA signaling network. Based on ABA-independent mechanisms, constitutively active receptors may be engineered and can be useful in the dissection of individual receptor function *in vivo* and in the generation of transgenic crops surviving environmental stresses (Hao et al., 2011; Mosquna et al., 2011; Li et al., 2013).

## Structural basis for SnRK2s kinases in the ABA signaling pathway

### Basal activity and autoactivation of SnRK2s kinases

The SnRK2s family members were identified from *wheat* (Anderberg and Walker-Simmons, 1992), fava *bean* (Li et al., 2000) and *Arabidopsis* (Mustilli et al., 2002; Yoshida et al., 2002). The SnRK2s family contains 10 members in *Arabidopsis*, among which the subclass III SnRK2s (SRK2D/SnRK2.2, SRK2E/OST1/SnRK2.6 and SRK2I/SnRK2.3) are strongly responsive to ABA (Yoshida et al., 2006). Several *snrk2s* mutants, such as the decuple mutant *snrk2.1/2/3/4/5/6/7/8/9/10* (=*srk2g/d/e/a/h/e/f/c/j/b*), the triple mutant *snrk2.2/3/6* (=*srk2d/e/i*) and the septuple mutant *snrk2.1/4/5/7/8/9/10* (=*srk2g/a/h/f/c/j/b*), completely preclude ABA responses, exhibiting conspicuously decreased tolerance to drought and dramatically increased insensitivity to ABA (Fujii and Zhu, 2009; Fujita et al., 2009) and a loss of dormancy and an ascent of seed ABA content compared to those of the wild-type (Fujita et al., 2009; Nakashima et al., 2009a; Fujii et al., 2011). The physiological functions of SnRK2s include transcriptional regulation, rapid regulations of stomatal closure (Pilot et al., 2001; Mustilli et al., 2002; Yoshida et al., 2002; Negi et al., 2008; Vahisalu et al., 2008; Geiger et al., 2009; Sato et al., 2009; Sirichandra et al., 2009; Chen et al., 2010; Kim et al., 2010), and response to ABA in seeds and vegetative tissues (Fujii et al., 2007). SnRK2s can also be activated by hyperosmotic stress (Mikolajczyk et al., 2000; Monks et al., 2001; Umezawa et al., 2004) in an activation pattern that is different from that of ABA (Boudsocq et al., 2004; Kobayashi et al., 2004). In particular, SnRK2.6 is important for the ABA-induced stomata closure in response to drought. SnRK2.2 and SnRK2.3 are predominantly responsible for the inhibition of seed germination and seedling growth in response to ABA. Subclass III SnRK2s contain a well-conserved catalytic domain and two conserved motifs. One motif is a highly acidic C-terminal segment that is termed ABA box, and the other one is a SnRK2 box that is important for kinase activity.

Upon ABA binding, PYLs undergo conformational changes to contact and inhibit PP2Cs (Melcher et al., 2009; Miyazono et al., 2009; Nishimura et al., 2009; Santiago et al., 2009a; Yin et al., 2009; Zhang et al., 2012). The inhibited PP2Cs result in the autoactivation of SnRK2s, which allows SnRK2s to relay the ABA signal (Cutler et al., 2010; Hubbard et al., 2010; Ng et al., 2011; Soon et al., 2012). Recently, the crystal structures of *Arabidopsis* SnRK2.3 and SnRK2.6 were reported (Ng et al., 2011) (Figures 4C–F). SnRK2.3 and SnRK2.6 kinases displayed a partially active state of phosphorylation-independent activity that can be activated by ABA intervention. Moreover, this partially active state can be extremely augmented by the phosphorylation of the activation loop by both intermolecularly (in *trans*) and intramolecularly (in *cis*) (Lochhead, 2009). Both SnRK2.3 and SnRK2.6 structures have the canonical kinase folds that are similar to those of AMPK and the yeast homolog Snf1 (Nayak et al., 2006; Chen et al., 2009; Littler et al., 2010) (Figures 4C,D). Compared to SnRK2.3, the SnRK2.6 structure seems to be more stable and contains more structural elements, including parts of the activation loop and the linker between the kinase domain and the SnRK2 box (Figure 4D). A noticeable feature is the SnRK2 box, containing a single α-helix that establishes extensive interactions with the αC helix. The intramolecular SnRK2s box-αC helix interaction, structurally resembling the intermolecular stabilization of the αC helix in Cdk2 by the helix α5 in cyclin (Jeffrey et al., 1995; Pavletich, 1999), is important for kinase activity. The active conformation structure of Pim-1, complexed with substrate peptide and the ATP analog AMP-PNP (Bullock et al., 2005), was superimposed with SnRK2.6 or SnRK2.3 to gain further insight into the mechanism of autophosphorylation. The detailed information about the two-step activation mechanism of SnRK2s kinases is also seen in the reference (Ng et al., 2011).

Simultaneously, two crystal structures of SnRK2.6 mutants (D160A and D160A/S175D) were reported, displaying an open inactive conformation and showing that the ABA-independent regulation motif (DI) stabilizes the conformation of the catalytically essential kinase αC helix (Yunta et al., 2011). The crystal structures of two catalytically inactive SnRK2.6 mutants are similar to the open, inactive conformation of the wild type (Ng et al., 2011).

The structures in combination with biochemical studies of SnRK2.6 and SnRK2.3 clarify the molecular basis of autophosphorylation activation of SnRK2s, which provides a complete structural framework for understanding the ABA-independent and -dependent regulations for a double-negative regulatory system (PYLs—|PP2C—|SnRK2).

### SnRK2s mimicking PYLs dock into PP2Cs

There are several lines of evidence of the interaction between SnRK2s and PP2Cs. Yeast two-hybrid analysis demonstrated that ABI1 physically contacted SnRK2.6 (Yoshida et al., 2006). Group A PP2Cs directly inactivate subclass III SnRK2s *in vitro* by dephosphorylating multiple serine/threonine residues in the kinase activation loop (Umezawa et al., 2009). For example, HAB1 dephosphorylates the kinase within this activation domain to repress the kinase activity (Belin et al., 2006; Boudsocq et al., 2007; Vlad et al., 2009). The S175 residue of SnRK2.6 was identified as a target site of PP2Cs by screening substrates of HAB1 or HAB^G246D^ (Vlad et al., 2009). Collectively, in the absence of ABA, SnRK2s are inactivated by PP2Cs (Ma et al., 2009; Park et al., 2009; Umezawa et al., 2009; Yin et al., 2009), which physically bind SnRK2s kinases and dephosphorylate a key serine in the kinase activation loop (Belin et al., 2006; Boudsocq et al., 2007; Vlad et al., 2009).

Recently, the solved SnRK2.6-HAB1 complex structure showed the mutual packing of both the kinase and phosphatase active sites, which formed the major binding interface (Soon et al., 2012) (Figure 4G). SnRK2.6 contributes three separate regions within the kinase domain for binding to HAB1. First, the activation loop inserts deeply into the catalytic cleft of HAB1 and mimics the “gate” loop of PYLs. In addition, the region near residues R139, I183 and E144 of SnRK2.6 emulates the cleft that is formed by the “gate” and “latch” loops of PYLs. The conserved tryptophan (such as HAB1 W385) from PP2Cs inserts into this region. In addition, the SnRK2.6 αG helix binds to the region near the PYLs interaction site in HAB1 (Figure 4G). The SnRK2s-PP2Cs binding interface largely overlaps with that of PYLs-PP2Cs, and the HAB1 structure in the SnRK2.6-HAB1 complex is nearly identical to that in the PYLs-HAB1 complex (Figure 4H). Thus, there is a marked similarity in PP2Cs recognition by SnRK2s and ABA receptors. Both SnRK2.6 and PYLs use a similar gate-and-lock mechanism to recognize PP2Cs (Soon et al., 2012) (Figure 4H). In addition to the kinase domain, the highly acidic C-terminal ABA box of SnRK2s is critical for interaction with PP2Cs (Boudsocq et al., 2004; Kobayashi et al., 2004; Yoshida et al., 2006). However, the ABA box has no clear electron density in the SnRK2.6-HAB1 complex structure. Mutagenesis and hydrogen/deuterium exchange experiments indicated that the negatively charged ABA box interacts primarily with the positively charged PP2Cs surface (Soon et al., 2012).

The SnRK2.6–HAB1 structure suggests a two-step mechanism by which HAB1 completely inactivates SnRK2.6. The first step is mediated by the catalytic activity of HAB1, which dephosphorylates S175 in the activation loop, thus reducing SnRK2.6 activity to the basal level. The second step is the physical inhibition of the SnRK2.6 kinase domain by HAB1 (Soon et al., 2012).

In *Arabidopsis*, there are 14 members of PYLs, nine members of PP2Cs and 10 members of SnRK2s, and differential binding between PP2Cs and PYLs, PP2Cs and SnRK2s is important for regulating the physiological responses of *Arabidopsis* to adapt to stress conditions.

## Application of the studies of ABA receptors for agriculture

Plant growth can be severely and constantly challenged by adverse environmental stresses, such as drought, salinity, and temperature fluctuations. Drought is one of the major abiotic stresses, and high salinity is the most severe environmental stress. Both of these stresses are closely related and interfere with plant growth in the overlapped mechanisms. Freezing injury leads to physical damages to tissues at temperatures below 0°C due to the formation of ice crystals in plant cells. These abiotic stresses adversely affect plant growth and development, reduce productivity and cause significant crop losses (Thomashow, 1999; Marris, 2008; Battisti and Naylor, 2009; Hubbard et al., 2010; Nakashima and Yamaguchi-Shinozaki, 2013; Roychoudhury et al., 2013).

Given these disadvantages of stresses, plants in response to environmental cues are being exploited to improve crop yield and reduce economic losses. First, the up- or down-regulation of some ABA signaling factors may improve tolerance to environmental stresses. Transgenic plants that over-express AREB/ABFs, such as DREB1A and OsbZIP23, showed improved drought tolerance (Xiang et al., 2008; Nakashima et al., 2009b; Fujita et al., 2011). In addition, the molecular mechanisms and wealth of structural information of the core ABA signaling pathway supply an approach of engineering the components, such as PYLs or PP2Cs. A recent study showed that the triple mutant (PYR1^H60P/V83F/F159V^) and the quadruple mutant (PYR1^H60P/V83F/M158I/F159V^), which were indistinguishable from ABA-saturated PYR1, stabilized their agonist-bound conformation to activate ABA signaling *in vivo* (Mosquna et al., 2011). The engineered HAB1^W385A^ mutant could modulate ABA signaling *in vivo* through the constitutive inactivation of the kinase SnRK2.6 even in the presence of ABA and PYLs (Dupeux et al., 2011a). In addition, the synthetic ABA analogs mimic ABA, which could control plant growth and development. (−)-ABA (Walker-Simmons et al., 1992; Nambara et al., 2002; Huang et al., 2007; Sirichandra et al., 2009; Zhang et al., 2013) and pyrabactin or its analogs (Park et al., 2009; Cutler et al., 2010; Hao et al., 2010; Melcher et al., 2010a; Peterson et al., 2010; Yuan et al., 2010; Mosquna et al., 2011; Zhang et al., 2012) had profound effects on plant growth, such as seed germination (Zhao et al., 2007). Last but not least, exploiting other plant growth regulators, ABA synthesis or an equivalent compound may prevent a decline in crop production and agricultural economy due to environmental stress.

## Conclusion and perspectives

The accumulating evidence indicate that PYLs function as *bona fide* ABA receptors, converging all aspects of ABA signaling. PYLs-mediated ABA signaling could play a crucial role in favoring stress adaptation and growth development for plants. ABA perception by PYLs receptors is orchestrated by PP2Cs and SnRK2s to establish a double-negative regulatory system for core ABA signaling, which controls ABA signaling in rapid stomatal closure responses for guard cells, as well as in long distance at the transcriptional level for seeds and vegetative tissues in response to water deficit. The detailed structural analyses, including ABA binding to PYLs, ABA-bound PYLs inhibiting PP2Cs and the ABA-induced inhibition of PP2Cs leading to the autoactivation of SnRK2 kinases, explain the gate-latch-lock mechanism (Melcher et al., 2009) and the two-step activation mechanism of SnRK2s kinases (Ng et al., 2011). In addition, the structural examination of SnRK2.6-HAB1 binary complex elucidated the two-step inactivation mechanism of SnRK2s by PP2Cs (Soon et al., 2012). These structural results agree with a substantial body of findings from biochemistry, molecular biology, and genetics. Although substantial progress has occurred in recent years, many questions remain. For example, why does the ABA signaling pathway in *Arabidopsis* involve 14 members in the PYLs family? What is the structural mechanism of individual receptors in their sensitivity and specialty in response to ABA? How can we exploit new selective ABA agonists and antagonists to ameliorate crop and ornamental plants? Finally, this review is just the beginning of understanding the ABA signaling pathway, and more work is required to gain global mechanistic insight into the complete signaling network.

## Summary

This review summarizes the structural information of multiple regulatory mechanisms and ABA signal transduction pathways by PYLs. This structural information elucidates the ABA-responsive and constitutive inhibition mechanisms, signaling pathways of ABA and ABA analog perception by PYLs and provides a basis for the further design of selective ABA analogs and agricultural application.

### Conflict of interest statement

The authors declare that the research was conducted in the absence of any commercial or financial relationships that could be construed as a potential conflict of interest.

## Abbreviations

ABA: Abscisic acid
ABI1/2: ABA insensitive 1/2
ABRE: ABA-responsive element
ABF: ABRE-binding factor
TFs: transcription factors
HAB1/2: Homology to ABA 1/2
PYR1: Pyrabactin resistance 1
PYL: PYR1-like
RCAR1: Regulatory component of ABA receptor 1
SnRK2: SNF1-related protein kinase 2
PP2C: 2C-type protein phosphatase.
