# Supplementary material for: Structural basis and functions of abscisic acid receptors PYLs
Source: Front Plant Sci. 2015 Feb 19;6:88. doi: 10.3389/fpls.2015.00088 (PMC4333806; doi:10.3389/fpls.2015.00088)
Supplement: Supplementary file 1 [file Presentation1.PDF]

Supporting Information

Figure S1. 2D map of interaction network between PYL3 and (+)-ABA. The hemispheres represent hydrophobic interactions, while the dotted lines represent polar interactions. The water molecules that are involved in hydrogen bonds and Mg^{2+} are shown in cyan and magenta spheres, respectively.

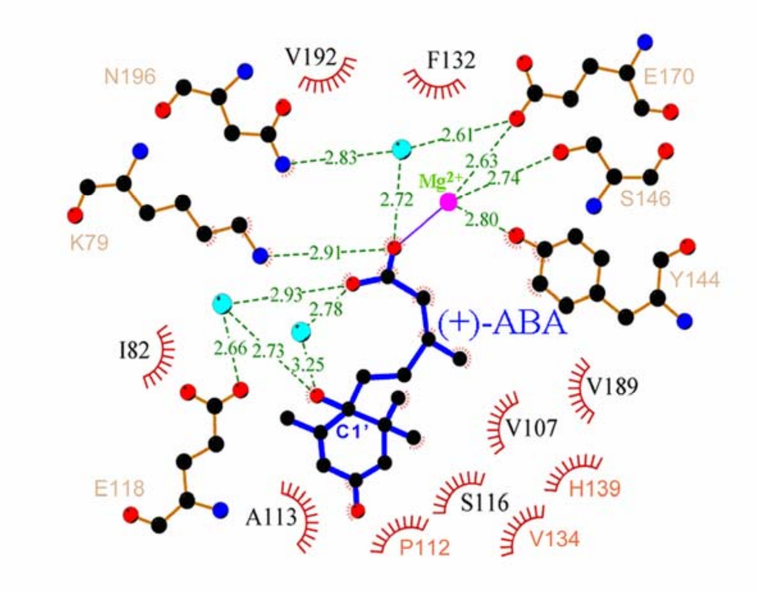

Figure S2. Secondary structures alignment of PYLs. All residues with similarities in physico-chemical properties are shown in yellow background with black character. The identical residues in red background are represented in white characters. The secondary structures of PYL3 are shown above the amino acid sequence, consisting of four helices and seven β strands. The nomenclature according to the previous publications is colored blue, for example, the L4 loop is also known as ‘gate’ (Melcher et al., 2009) or CL2 (Yin et al., 2009) and the L5 loop is also known as ‘latch’ (Melcher et al., 2009) or CL3 (Yin et al., 2009), whose corresponding residues are emphasized by blue dotted rectangles. The critical residues in PYLs coordinating the ABA are marked with triangles filled green (see Figure 2). Three variable residues responsible for the stereospecificity of PYLs to ABA enantiomers are labeled with rectangles filled green cyan (see Figure 3B&C). The critical residues in PYLs involved in HABI1 interactions are marked with triangles filled black (see Figure 4A&B). The residue involved in PYLs constitutive binding and inhibition of PP2Cs is

marked with triangles filled red (see Figure 5). The sequence alignment is generated by ClustalW (Larkin et al., 2007).

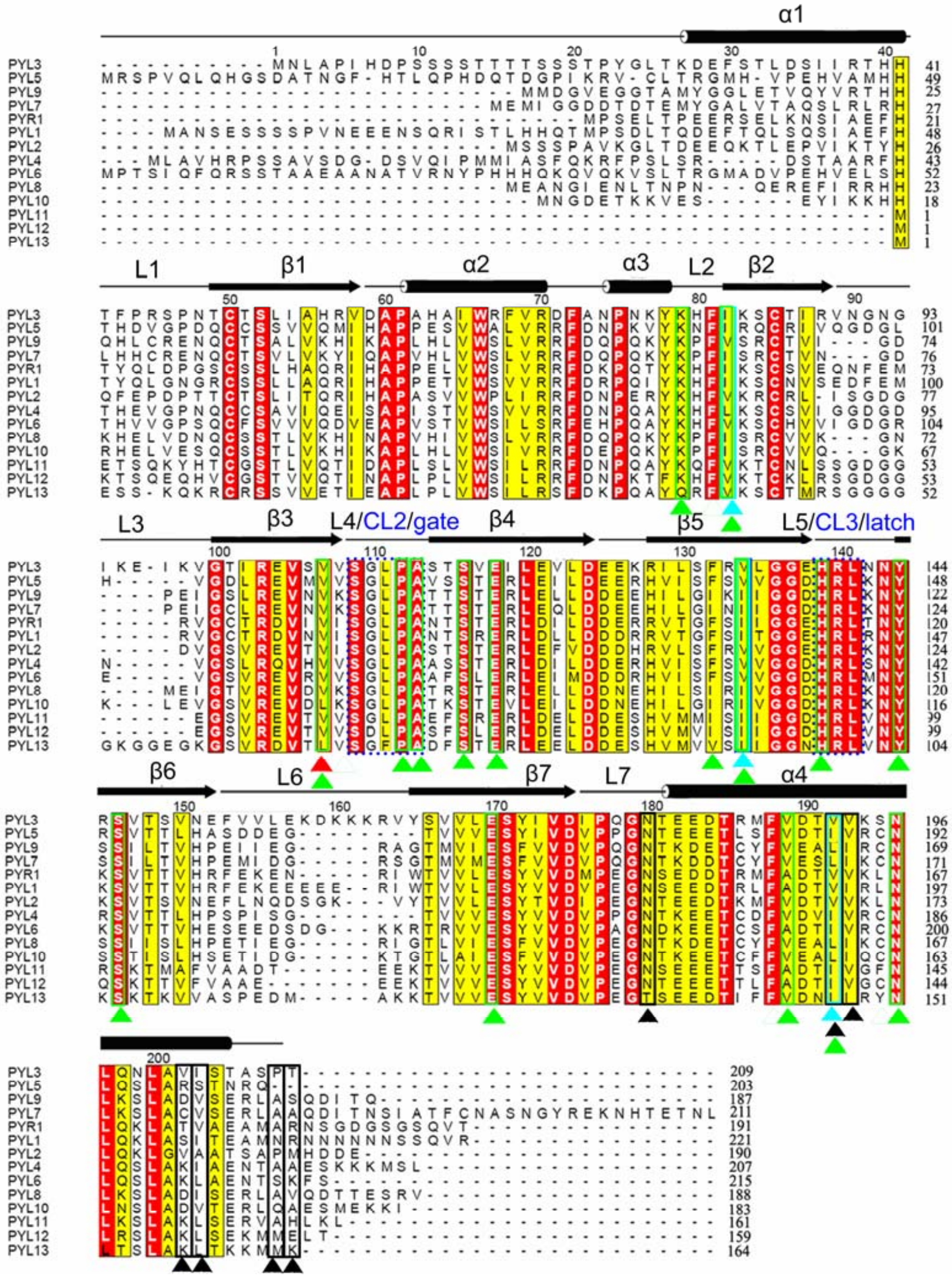

Reference:

Larkin, M.A., Blackshields, G., Brown, N.P., Chenna, R., Mcgettigan, P.A., Mcwilliam, H., et al. (2007). Clustal W and Clustal X version 2.0. *Bioinformatics* 23**,** 2947-2948.

doi:10.1093/bioinformatics/btm404

Melcher, K., Ng, L.M., Zhou, X.E., Soon, F.F., Xu, Y., Suino-Powell, K.M., et al. (2009). A gate-latch-lock mechanism for hormone signalling by abscisic acid receptors. *Nature* 462**,** 602-608. doi:10.1038/nature08613

Yin, P., Fan, H., Hao, Q., Yuan, X., Wu, D., Pang, Y., et al. (2009). Structural insights into the mechanism of abscisic acid signaling by PYL proteins. *Nature structural & molecular biology* 16**,** 1230-1236. doi:10.1038/nsmb.1730
